# A dyad approach to understanding relationship satisfaction and health outcomes in military couples following service member and veteran traumatic brain injury

**DOI:** 10.3389/fpsyt.2025.1465801

**Published:** 2025-03-12

**Authors:** Tracey A. Brickell, Brian J. Ivins, Megan M. Wright, Jamie K. Sullivan, Louis M. French, Rael T. Lange

**Affiliations:** ^1^ Traumatic Brain Injury Center of Excellence, Walter Reed National Military Medical Center, Bethesda, MD, United States; ^2^ National Intrepid Center of Excellence, Walter Reed National Military Medical Center, Bethesda, MD, United States; ^3^ Uniformed Services University of the Health Sciences, Bethesda, MD, United States; ^4^ General Dynamics Information Technology, Silver Spring, MD, United States; ^5^ Traumatic Brain Injury Center of Excellence, Silver Spring, MD, United States; ^6^ CICONX, Silver Spring, MD, United States

**Keywords:** service member, veteran, couple, intimate partner, traumatic brain injury, relationship satisfaction, dyad

## Abstract

**Objective:**

Using a dyadic approach, this study examined health and family outcomes in military couples following service member and veteran (SMV) traumatic brain injury (TBI), within the context of relationship satisfaction.

**Methods:**

Participants included 164 dyads (*N* = 328), composed of US SMVs (*n* = 164) and their intimate partners (IPs, *n* = 164). Dyads completed a measure of relationship satisfaction, as well as measures of psychological, social, caregiving, family, neurobehavioral, and/or PTSD outcomes. Dyads were classified into four relationship satisfaction groups: (1) SMV and IP satisfied (Both Satisfied, *n* = 72 dyads), (2) SMV satisfied and IP dissatisfied (SMV_sat_/IP_dis_, *n* = 25 dyads), (3) SMV dissatisfied and IP satisfied (SMV_dis_/IP_sat_, *n* = 21 dyads), and (4) SMV and IP dissatisfied (Both Dissatisfied, *n* = 46 dyads).

**Results:**

Within dyads, SMVs reported worse scores than their IPs, except in the SMV_sat_/IP_dis_ group, where their dissatisfied IPs reported worse scores on four measures. Across groups, dissatisfied SMVs reported worse scores compared to satisfied SMVs, and dissatisfied IPs reported worse scores compared to satisfied IPs. Satisfied and dissatisfied SMVs and IPs in the mixed relationship satisfaction groups reported little to no differences across measures compared to their respective SMVs and IPs in the Both Satisfied and Both Dissatisfied groups, with the exception of the family measures for dissatisfied SMVs or IPs.

**Conclusions:**

Relationship dissatisfaction was related to worse health and family outcomes, even when the other members of the dyad reported satisfaction in their relationship. A dual-goal, dyadic approach to TBI treatment that focuses on how individual, couple, and family factors interact will likely maximize service member recovery and return to duty, as well as outcomes for military families.

## Introduction

Traumatic brain injury (TBI) is common among military service members. Over 80% of TBIs sustained in the military are classified as mild TBI (MTBI) (1). Long-term complications are less likely following a MTBI compared to moderate, severe, or penetrating TBI. In the military, training accidents and combat experiences often occur both during and pre- and post-TBI, contributing to the development of physical and psychological comorbid conditions. The symptom profile of many co-occurring clinical conditions often overlaps with neurobehavioral symptoms and can result in significant disruption to physical, psychological, and social functioning in service members and veterans (SMV) following a TBI of any severity (2).

SMVs with chronic neurobehavioral symptoms often require ongoing care, support, and advocacy from family members, most frequently their intimate partners (IPs) (3–6). Military family caregivers generally lack formal medical education or training to manage neurobehavioral and comorbid symptoms, often leaving them unprepared and overwhelmed (7). Regardless of TBI severity (3), care provision related to the SMV’s neurobehavioral and comorbid symptoms has consistently been associated with poor physical, psychological, social, and caregiving health-related quality of life (HRQOL) in military family caregivers (4, 6, 8–11). As part of the 15-year longitudinal Caregiver and Family Member Study (CGFM Study)^1^, our team examined outcomes in military family members specifically across SMV TBI severity (3). Worse HRQOL in family members was associated with SMVs who had a remote uncomplicated MTBI, compared to those with a more severe TBI (complicated mild, moderate, severe, or penetrating). Given the average time since MTBI was 11.3 years, the authors concluded that any TBI effects were likely interrelated with comorbid symptoms (Posttraumatic stress disorder (PTSD), depression, headaches, and chronic pain). IPs commonly report changes in the dynamics of their relationship with the SMV, navigating both care provision and romantic roles (12). Higher levels of caregiving distress have been associated with lower relationship satisfaction and divorce considerations in IPs of injured SMVs (13, 14).

Research examining relationship satisfaction in military couples has largely focused on understanding how PTSD symptoms impact, and are impacted by, relationship satisfaction (15). PTSD symptoms are categorized into four symptom clusters, including (1) intrusive thoughts or re-experiencing the traumatic event (cluster B: intrusion); (2) persistent avoidance of stimuli associated with the traumatic event (cluster C: avoidance); (3) overly negative thoughts and feelings (cluster D: negative thinking and mood); and (4) increased arousal, anger, and irritability (cluster E: hyperarousal). When all PTSD symptom clusters were examined simultaneously, cluster D symptoms generally accounted for the greatest variance in relationship distress, followed by clusters C and E, but not cluster B (15). IPs often accommodate their emotions and behaviors in an attempt to manage or reduce the SMV’s PTSD symptoms, such as avoiding contentious conversations, intimacy, social situations, and household noise, as well as assuming household chores, roles, and responsibilities previously shared with the SMV (15, 16). While often well-intentioned, accommodative behavioral and emotional actions can lead to elevated IP psychological, social, caregiving, relationship, and family distress. Accommodations can also inadvertently reinforce or facilitate PTSD symptoms, undermine treatment goals, and impede recovery and fitness-for-duty outcomes.

IPs of SMVs with TBI often describe engaging in similar behavioral and emotional accommodative actions and experiencing heightened psychological, social, caregiving, relationship, and family distress (12, 17). PTSD is a common comorbid condition in SMVs with TBI and has been associated with chronic neurobehavioral self-reported symptoms up to 10 years following a TBI of any severity (18–20). Our team examined health outcomes and family disruption within the context of relationship satisfaction in IPs providing care and support to SMVs with concurrent MTBI and PTSD (14). Nearly half of IPs reported experiencing dissatisfaction in their relationship with the SMV. IPs who reported greater relationship dissatisfaction also reported higher levels of caregiving strain, anxiety, and vigilance; more feelings of rejection, loss, and being trapped; less companionship and emotional support; more social isolation; and greater family disruption. Lower levels of relationship satisfaction were associated with SMV neurobehavioral symptoms related to adjustment (e.g., mood, anxiety, pain, headaches, fatigue, aggression, social/family relationships), but not with those related to ability (e.g., mobility, communication, attention/concentration, memory, visual). Our team also examined health outcomes in family members within the first 12 months after identifying that they were no longer providing care to the SMV (21). No longer being in an intimate relationship with the SMV was one of the most frequently endorsed reasons for ceasing caregiving. Compared to family members who were still providing care, those who were no longer caregiving were more likely to report dissatisfaction in both their intimate and caregiving relationships during the caregiving period. A limitation of our previous research was the reliance on IP reports only. Relying solely on IP reports fails to consider the perspectives of both partners in the relationship.

Using a dyadic approach, the current study builds on previous research with military couples and SMV TBI by exploring psychological, social, caregiving, family, neurobehavioral, and/or PTSD outcomes in intimate dyads within the context of relationship satisfaction.

## Materials and methods

### Participants

Participants included 164 dyads (*N* = 328) enrolled in the CGFM Study, comprising US SMVs (*n* = 164) with a TBI diagnosed at a Department of Defense or Veterans Affairs (DoD/VA) treatment facility, and their IPs (*n* = 164). Recruitment procedures targeted the IPs, with SMVs invited to participate through their IPs. Over 80% of IPs were recruited from nationwide publicity via social media and at events. The remainder were recruited during a National Intrepid Center of Excellence (NICoE) family orientation session at the Walter Reed National Military Medical Center (WRNMMC), while the service member was receiving treatment for MTBI in the interdisciplinary intensive outpatient program (IOP). Dyads were included in the current study if they provided signed informed consent, were 18 years or older, and were fluent in English. Study procedures and recruitment materials were conducted in accordance with the Institutional Review Board of WRNMMC and the guidelines of the Declaration of Helsinki. Sample characteristics are presented in **Table 1**.

**Table 1:** Intimate partner and service member or veteran characteristics.

Service Member or Veteran	
Male (*n*; %)	161 (98.2)
Age in years (M; SD)	44.9 (7.3)
Veteran^a^ (*n*; %)	139 (84.8)
Years since Traumatic Brain Injury^b,c^ (M; SD)	12.4 (5.6)
Uncomplicated Mild Traumatic Brain Injury^b,c^ (*n*; %)	139 (84.8)
Posttraumatic Stress Disorder^c^ (*n*; %)	127 (77.4)
Depression^c^ (*n*; %)	78 (47.6)
Substance-related^c^ (*n*; %)	31 (18.9)
Chronic Headaches^c^ (*n*; %)	118 (72.0)
Chronic Pain^c^ (*n*; %)	111 (67.7)
Sleep^c^ (*n*; %)	75 (45.7)
Significant Bodily Injury^c,d^ (*n*; %)	3 (1.8)
Intimate Partner	
Female (*n*; %)	161 (98.2)
Age in years (M; SD)	43.8 (7.4)
White (*n*; %)	37 (22.6)
Employed^e^ (*n*; %)	88 (53.7)
Household Income < 40,000^f^ (*n*; %)	8 (4.9)
Providing ongoing care and support^g^ (*n*; %)	150 (91.5)
Parenting children^h^ (*n*; %)	99 (60.4)

*N* = 328 (164 SMVs, 164 IPs).

*SMV*, service member and veteran; *IP*, intimate partner; *TBI*, traumatic brain injury.

a Army, 54.9%; Marine Corps, 14.6%; Navy, 20.1%; Air Force, 9.1%; Coast Guard, 0.6%; Special Operations Forces, 9.1%; one participant missing response.

b Complicated mild TBI, 2.4%; moderate TBI, 3.0%; severe TBI, 3.7%; penetrating TBI, 6.1%. Calculated based on the most severe TBI. If there were multiple TBIs with the same severity that met the criteria for the most severe, the most recent injury was chosen.

c Diagnosis obtained from medical records.

d Examples: skull fracture, spinal cord injury, burns to eye/ear, internal organs, or orthopedic/amputation.

e Working > 24 h/week, 68.2%.

f 2024 US Department of Health and Human Services Federal poverty level for four to five persons in a family/household = $31,200–$36,580.

g Years caregiving: M = 9.5 (SD = 4.8); 48.0% caregiving > 6 h/day.

h M = 2.1 (SD = 1.2) children per dyad.

### Measures and procedure

Dyads completed questionnaires between April 2020 to September 2023 through telephone/web-based procedures from a remote location during a scheduled appointment with a study investigator on the telephone, who addressed any administration issues and quality control procedures. The completion of the questionnaires was self-directed by the SMV or IP and occurred during the same appointment, but independently of each other.

#### IP and SMV dyad measures

Dyads completed the Couples Satisfaction Index (CSI-4) (22) four-item short-form measure. Items were summed to create a total score, with higher scores reflecting greater relationship satisfaction. Dyads were classified into satisfied (≥ 13.5, SMV: *n* = 97, IP: *n* = 93) or dissatisfied (< 13.5, SMV: *n* = 67 IP: *n* = 71) relationship categories using a cut-score recommended by the test developers. Dyads were further classified into four relationship satisfaction dyad groups as follows: (1) SMV and IP satisfied (Both Satisfied, *n* = 72 dyads), (2) SMV satisfied and IP dissatisfied (SMV_sat_/IP_dis_, *n* = 25 dyads), (3) SMV dissatisfied and IP satisfied (SMV_dis_/IP_sat_, *n* = 21 dyads), and (4) SMV and IP dissatisfied (Both Dissatisfied, *n* = 46 dyads).

Dyads completed seven HRQOL (23) short-form measures reflecting psychological HRQOL (Anxiety, Depression, Anger, General Life Satisfaction) and social HRQOL (Ability to Participate in Social Roles and Activities, Social Isolation, Emotional Support, Perceived Rejection). A total raw score for each scale was calculated and converted to a T-score (M = 50, SD = 10) using established conversion tables. Ability to Participate in Social Roles and Activities, Emotional Support, and General Life Satisfaction were recorded such that higher scores reflected worse functioning for all measures.

Two measures assessing family relationships were also completed by dyads. The Family Assessment Device General Functioning subscale (FAD-GF) (24) measures family functioning. In the absence of established T-scores, a total raw score was calculated and converted to a T-score using the mean and standard deviation (M = 1.66, SD = 0.47) from an independent sample of US SMVs and IPs (25). The Deployment Risk and Resilience Inventory-2 Postdeployment Family Experiences measures the quality of postdeployment family relationships in terms of communication and closeness among family members. In the current study, the wording “after your most recent deployment” was removed from the instructions to assess family experiences more generally. In the absence of established T-scores, a total raw score was calculated and converted to a T-score using the mean and standard deviation from an independent sample of US SMVs (M = 47.57, SD = 11.49) (26).

#### SMV-only measures

SMVs additionally completed the Neurobehavioral Symptom Inventory (NSI) (27) measure of postconcussion symptoms. In the absence of established T-scores, a total raw score was calculated and converted to a T-score using the mean and standard deviation from an independent sample of US SMVs (M = 10.8, SD = 11.3) (28). The PTSD Checklist for DSM-5 (PCL-5) (29) was completed to assess PTSD symptoms per the Diagnostic and Statistical Manual of Mental Disorders Fifth Edition (DSM-5). DSM-5 symptom cluster scores were obtained by summing the scores for the items within a given cluster as follows: cluster B (re-experiencing, items 1–5), cluster C (avoidance, items 6 and 7), cluster D (negative cognitions and mood, items 8–14), and cluster E (arousal, items 15–20). In the absence of established T-scores, cluster scores were converted to a T-score using the mean and standard deviation from an independent sample of US SMVs (cluster B: M = 9.28, SD =5.87; cluster C: M = 4.06, SD = 2.60; cluster D: M = 12.54, SD = 8.15; and cluster E: M = 11.09, SD = 6.75 (30).

#### IP-only measures

IPs completed an additional three caregiving-related HRQOL short forms (23), including Emotional Suppression, Caregiver-Specific Anxiety, and Caregiver Strain. A total raw score for each scale was calculated and converted to a T-score using established conversion tables. The 13-item Caregiving Relationship Satisfaction subscale from the Caregiver Appraisal Scale (31) was summed according to Brickell and colleagues (32) and converted to a T-score using the mean and standard deviation from the same study (M = 50.80, SD = 8.16). T-scores were transposed so that higher scores reflected worse functioning for all measures.

#### Sample characteristics

Sample characteristics in **Table 1** were provided by IPs. A review of the SMV’s DoD/VA medical records was conducted for information on TBI and comorbid diagnoses.

### Statistical analysis plan

First, repeated measures ANOVA was used to compare scores between SMVs and IPs within dyads on the HRQOL and family measures that both members of the dyad completed, across the four relationship satisfaction dyad groups: (1) Both Satisfied, (2) SMV_sat_/IP_dis_, (3) SMV_dis_/IP_sat_, and (4) Both Dissatisfied. Second, ANOVA was used to compare SMV scores on the psychological and social HRQOL, family, NSI, and PCL-5 measures across the relationship satisfaction dyad groups. Third, IP scores on the psychological, social, and caregiving HRQOL, as well as family measures, were compared across the relationship satisfaction dyad groups using ANOVA. Cohen’s effect size *d* was calculated for each pairwise comparison, with the interpretations as follows: small = 0.2, medium = 0.5, and large = 0.8. The Benjamini and Hochberg (33) step-down procedure was applied to control the false discovery rate for each family of ANOVA *f*-tests and pairwise comparisons. The Benjamini and Hochberg procedure is less conservative and more powerful than methods that control the familywise error rate, such as the Bonferroni correction, particularly when the number of multiple comparisons is large.

## Results

Within dyads, SMVs tended to report worse scores than their IPs (**Table 2**), with the exception of the SMV_sat_/IP_dis_ group, where their dissatisfied IPs reported worse scores on General Life Satisfaction, Emotional Support, Perceived Rejection, and Family Functioning measures (*p* = 0.003–0.024, *d* = 0.61–0.78). Dissatisfied SMVs in the SMV_dis_/IP_sat_ group reported worse scores on all measures compared to their IPs (*p* = < 0.001–0.009, *d* = 0.76–1.51). In the Both Satisfied group, SMVs reported worse scores on Anxiety, Depression, Anger, Ability to Participate in Social Roles and Activities, Social Isolation, and Family Experiences measures (*p* = < 0.001–0.023, *d* = 0.32–0.93) compared to their IPs. SMVs in the Both Dissatisfied group reported worse scores on Anxiety, Depression, Anger, General Life Satisfaction, Social Isolation, and Family Experiences (*p* = < 0.001–0.018, *d* = 0.41–1.26) compared to their IPs.

**Table 2:** Descriptive statistics for intimate partner and service member or veteran dyads by relationship satisfaction group.

Dyad measures	Dyad	Relationship satisfaction group															
		1. Both Satisfied				2. SMV_sat_/IP_dis_				3. SMV_dis_/IP_sat_				4. Both Dissatisfied			
		M	SD	*p*-value	*d*	M	SD	*p*-value	*d*	M	SD	*p*-value	*d*	M	SD	*p*-value	*d*
Anxiety	SM	57.7	9.5	< 0.001^b^	0.79	57.4	11.9	0.769	0.07	63.4	8.5	< 0.001^b^	1.25	62.5	7.7	< 0.001^b^	0.86
	IP	50.4	8.9			58.1	8.8			51.3	10.8			55.6	8.1		
Depression	SM	54.0	10.1	< 0.001^b^	0.78	56.2	11.0	0.900	0.04	62.2	9.0	< 0.001^b^	1.35	60.2	8.3	< 0.001^b^	0.80
	IP	46.9	8.2			55.8	8.0			49.1	10.4			54.0	7.3		
Anger	SM	56.8	9.6	< 0.001^b^	0.93	55.2	11.0	0.370	0.25	64.2	9.4	< 0.001^b^	1.51	61.9	9.5	< 0.001^b^	0.85
	IP	47.9	9.5			52.8	8.5			48.0	12.1			54.1	8.8		
General Life Satisfaction^a^	SM	47.1	10.5	0.087	0.26	50.3	9.1	0.024	0.61	55.9	8.5	< 0.001^b^	1.32	58.6	7.1	0.018^b^	0.41
	IP	44.8	7.6			55.3	7.1			45.8	6.9			55.7	6.9		
Social Role Activities^a^	SM	54.7	9.1	0.023^b^	0.32	56.0	7.7	0.542	0.16	61.3	7.2	< 0.001^b^	1.39	58.6	7.6	0.089	0.29
	IP	52.2	7.3			57.2	7.2			51.2	7.4			56.4	7.8		
Social Isolation	SM	52.3	9.2	0.020^b^	0.36	55.3	11.1	0.860	0.05	61.3	5.6	< 0.001^b^	1.22	57.9	8.2	0.009	0.53
	IP	49.2	7.8			54.8	7.8			51.5	10.6			54.1	6.0		
Emotional Support^a^	SM	46.3	7.2	0.190	0.20	48.5	7.2	0.013	0.77	56.5	6.6	0.006^b^	0.91	57.9	6.7	0.107	0.34
	IP	47.9	7.6			54.9	9.6			50.7	6.2			55.4	8.0		
Perceived Rejection	SM	49.0	10.3	0.396	0.14	50.8	12.6	0.005	0.72	61.3	12.8	0.009^b^	0.76	62.5	9.7	0.053	0.44
	IP	50.6	12.3			59.3	11.1			52.5	10.5			58.0	10.7		
Family Functioning	SM	48.8	9.3	0.366	0.14	55.0	13.8	0.003	0.78	58.9	11.9	0.002^b^	0.95	67.4	11.4	0.116	0.31
	IP	47.6	8.7			64.1	9.6			50.0	7.0			64.4	8.0		
Family Experiences	SM	46.4	6.6	< 0.001^b^	0.63	51.3	10.8	0.934	0.02	53.5	7.9	< 0.001^b^	1.26	58.6	9.3	< 0.001^b^	1.26
	IP	42.9	4.7			51.1	10.8			45.4	5.0			49.3	5.5		

*N* = 328 (164 SMVs, 164 IPs): Both Satisfied = 72 dyads; SMV_sat_/IP_dis_ = 25 dyads; SMV_dis_/IP_sat_ = 21 dyads; Both Dissatisfied = 46 dyads.

*IP*, intimate partner; *SMV*, service member or veteran; *Social role activities*, ability to participate in social roles and activities.

a Scores were transposed so that higher scores reflected worse functioning for all measures.

b Statistical significance after controlling the false discovery rate using the Benjamini and Hochberg (33) step-down procedure to adjust the significance criterion. Cohen’s *d* effect size interpretation *d*: small, 0.2; medium, 0.5; large, 0.8.

Across the relationship satisfaction dyad groups (**Table 3**), dissatisfied SMVs in the Both Dissatisfied and SMV_dis_/IP_sat_ groups reported worse scores on (1) all measures compared to SMVs in the Both Satisfied group (1v3, 1v4: *p* = < 0.001–0.040, *d* = 0.39–1.84), and (2) most measures compared to SMVs in the SMV_sat_/IP_dis_ group (2v4, 2v3: *p* = *<* 0.001–0.038, *d* = 0.54–1.37). Several meaningful effect sizes (*d* ≥ 0.40) were also found that did not reach significance (*p* ≥ 0.05), likely due to the small sample size for the SMV_dis_/IP_sat_ group. The only differences between SMVs who were satisfied in their relationship (1v2) and SMVs who were dissatisfied in their relationship (3v4) were in family functioning and family experiences measures (2 > 1; 4 > 3: *p* = 0.007–0.033, *d* = 0.57–0.74).

**Table 3:** Descriptive statistics for service member or veteran measures by relationship satisfaction group.

Service member and veteran measures	Relationship satisfaction group																				
	1		2		3		4		ANOVA	Pairwise comparisons											
	Both Satisfied		SMV_sat_/IP_dis_		SMV_dis_/IP_sat_		Both Dissatisfied			1v2		1v3		1v4		2v3		2v4		3v4	
	M	SD	M	SD	M	SD	M	SD	*p*-value	*p*-value	*d*	*p*-value	*d*	*p*-value	*d*	*p*-value	*d*	*p*-value	*d*	*p*-value	*d*
Anxiety	57.7	9.5	57.4	11.9	63.4	8.5	62.5	7.7	0.008^b^	0.901	0.03	0.015^b^	0.62	0.005^b^	0.55	0.058	0.58	0.032	0.55	0.660	0.12
Depression	54.0	10.1	56.2	11.0	62.2	9.0	60.2	8.3	0.001^b^	0.373	0.21	0.001^b^	0.83	0.001^b^	0.66	0.051	0.60	0.088	0.43	0.374	0.24
Anger	56.8	9.6	55.2	11.0	64.2	9.4	61.9	9.5	0.001^b^	0.495	0.16	0.002^b^	0.77	0.006^b^	0.53	0.005^b^	0.88	0.009^b^	0.67	0.361	0.24
General Life Satisfaction^a^	47.1	10.5	50.3	9.1	55.9	8.5	58.6	7.1	< 0.001^b^	0.177	0.32	0.001^b^	0.87	< 0.001^b^	1.25	0.038	0.63	< 0.001^b^	1.06	0.187	0.35
Social Role Activities^a^	54.7	9.1	56.0	7.7	61.3	7.2	58.6	7.6	0.006^b^	0.540	0.14	0.003^b^	0.76	0.018^b^	0.45	0.021	0.71	0.174	0.34	0.179	0.36
Social Isolation	52.3	9.2	55.3	11.1	61.3	5.6	57.9	8.2	< 0.001^b^	0.182	0.31	< 0.001^b^	1.08	0.001^b^	0.64	0.029	0.70	0.264	0.28	0.088	0.46
Emotional Support^a^	46.3	7.2	48.5	7.2	56.5	6.6	57.9	6.7	< 0.001^b^	0.213	0.29	< 0.001^b^	1.43	< 0.001^b^	1.64	< 0.001^b^	1.15	< 0.001^b^	1.37	0.425	0.21
Perceived Rejection	49.0	10.3	50.8	12.6	61.3	12.8	62.5	9.7	< 0.001^b^	0.478	0.17	< 0.001^b^	1.13	< 0.001^b^	1.34	0.008^b^	0.83	< 0.001^b^	1.09	0.669	0.11
Family Functioning	48.8	9.3	55.0	13.8	58.9	11.9	67.4	11.4	< 0.001^b^	0.013	0.60	< 0.001^b^	1.02	< 0.001^b^	1.84	0.316	0.30	< 0.001^b^	1.01	0.007^b^	0.74
Family Experiences	46.4	6.6	51.3	10.8	53.5	7.9	58.6	9.3	< 0.001^b^	0.009	0.64	< 0.001^b^	1.02	< 0.001^b^	1.59	0.453	0.23	0.004^b^	0.74	0.033	0.57
NSI	70.5	13.8	73.3	18.0	78.4	14.1	79.9	13.2	0.004^b^	0.418	0.19	0.023^b^	0.57	< 0.001^b^	0.70	0.304	0.31	0.087	0.44	0.680	0.11
PCL-5 cluster B	45.6	9.3	47.7	9.5	51.8	8.9	49.2	8.9	0.030^b^	0.335	0.23	0.008^b^	0.67	0.040^b^	0.39	0.139	0.45	0.515	0.16	0.260	0.30
PCL-5 cluster C	46.9	10.6	46.8	9.5	54.0	10.7	51.8	8.8	0.006^b^	0.983	0.01	0.009^b^	0.67	0.011^b^	0.49	0.021	0.71	0.032	0.54	0.378	0.23
PCL-5 cluster D	46.5	9.1	48.6	9.4	55.4	9.5	54.2	7.9	< 0.001^b^	0.316	0.23	< 0.001^b^	0.97	< 0.001^b^	0.90	0.020	0.71	0.009^b^	0.67	0.618	0.13
PCL-5 cluster E	48.8	8.4	49.6	9.9	54.4	8.0	54.2	7.2	0.002^b^	0.722	0.08	0.009^b^	0.67	< 0.001^b^	0.68	0.081	0.53	0.026^b^	0.57	0.946	0.02

*N* = 328 (164 SMVs, 164 IPs): Both Satisfied = 72 dyads; SMV_sat_/IP_dis_ = 25 dyads; SMV_dis_/IP_sat_ = 21 dyads; Both Dissatisfied = 46 dyads.

*SMV*, service member or veteran; *Social role activities*, ability to participate in social roles and activities; *NSI*, Neurobehavioral Symptom Inventory; *PCL-5*, PTSD Checklist for DSM-5 (cluster B: re-experiencing; cluster C: avoidance; cluster D: negative cognitions and mood; cluster E: arousal).

a Scores were transposed so that higher scores reflected worse functioning for all measures.

b Statistical significance after controlling the false discovery rate using the Benjamini and Hochberg (33) step-down procedure to adjust the significance criterion. Cohen’s *d* effect size interpretation *d*: small, 0.2; medium, 0.5; large, 0.8.

Across the relationship satisfaction dyad groups (**Table 4**), dissatisfied IPs in the Both Dissatisfied and SMV_sat_/IP_dis_ groups reported worse scores on (1) all measures compared to IPs in the Both Satisfied group (1v2, 1v4: *p* = < 0.001–0.024, *d* = 0.53–2.05), and (2) most measures compared to the SMV_dis_/IP_sat_ group (2v3, 3v4: *p* = < 0.001–0.038, *d* = 0.59–1.87). Several nonsignificant meaningful effect sizes were also found between dissatisfied IPs in both groups and the SMV_dis_/IP_sat_ group (2v3, 3v4: *p* ≥ 0.05, *d* ≥ 0.40). The Family Experiences measure was the only measure where satisfied IPs differed across groups (3>1: *p* = 0.040, *d* = 0.53). No differences were found between dissatisfied IPs across groups (2v4).

**Table 4:** Descriptive statistics for intimate partner measures by relationship satisfaction group.

Intimate partner measures	Relationship satisfaction group																				
	1		2		3		4		ANOVA	Pairwise comparisons											
	Both Satisfied		SMV_sat_/IP_dis_		SMV_dis_/IP_sat_		Both Dissatisfied			1v2		1v3		1v4		2v3		2v4		3v4	
	M	SD	M	SD	M	SD	M	SD	*p*-value	*p*-value	*d*	*p*-value	*d*	*p*-value	*d*	*p*-value	*d*	*p*-value	*d*	*p*-value	*d*
Anxiety	50.4	8.9	58.1	8.8	51.3	10.8	55.6	8.1	< 0.001^b^	< 0.001^b^	0.88	0.677	0.10	0.001^b^	0.62	0.023^b^	0.70	0.235	0.30	0.072	0.49
Depression	46.9	8.2	55.8	8.0	49.1	10.4	54.0	7.3	< 0.001^b^	< 0.001^b^	1.10	0.306	0.26	< 0.001^b^	0.91	0.018^b^	0.73	0.339	0.24	0.030^b^	0.59
Anger	47.9	9.5	52.8	8.5	48.0	12.1	54.1	8.8	0.003^b^	0.024^b^	0.53	0.954	0.01	0.001^b^	0.67	0.124	0.47	0.553	0.15	0.023^b^	0.62
General Life Satisfaction^a^	44.8	7.6	55.3	7.1	45.8	6.9	55.7	6.9	< 0.001^b^	< 0.001^b^	1.40	0.603	0.13	< 0.001^b^	1.48	< 0.001^b^	1.36	0.841	0.05	< 0.001^b^	1.44
Social Role Activities^a^	52.2	7.3	57.2	7.2	51.2	7.4	56.4	7.8	0.001^b^	0.004^b^	0.69	0.596	0.13	0.003^b^	0.57	0.008^b^	0.82	0.690	0.10	0.012^b^	0.68
Social Isolation	49.2	7.8	54.8	7.8	51.5	10.6	54.1	6.0	0.002^b^	0.003^b^	0.71	0.292	0.26	< 0.001^b^	0.69	0.228	0.37	0.693	0.10	0.193	0.36
Emotional Support^a^	47.9	7.6	54.9	9.6	50.7	6.2	55.4	8.0	< 0.001^b^	< 0.001^b^	0.87	0.122	0.39	< 0.001^b^	0.98	0.087	0.53	0.817	0.06	0.019^b^	0.64
Perceived Rejection	50.6	12.3	59.3	11.1	52.5	10.5	58.0	10.7	0.001^b^	0.002^b^	0.73	0.520	0.16	0.001^b^	0.64	0.038	0.63	0.632	0.12	0.051	0.52
Family Functioning	47.6	8.7	64.1	9.6	50.0	7.0	64.4	8.0	< 0.001^b^	< 0.001^b^	1.86	0.250	0.29	< 0.001^b^	2.00	< 0.001^b^	1.68	0.906	0.03	< 0.001^b^	1.87
Family Experiences	42.9	4.7	51.1	10.8	45.4	5.0	49.3	5.5	< 0.001^b^	< 0.001^b^	1.31	0.040	0.52	< 0.001^b^	1.27	0.030^b^	0.71	0.344	0.25	0.007^b^	0.74
Emotional Suppression	44.5	10.5	51.1	10.4	45.5	12.8	51.0	9.2	0.003^b^	0.008^b^	0.63	0.702	0.10	0.001^b^	0.64	0.108	0.49	0.939	0.02	0.054	0.52
Caregiver Strain^c^	48.3	7.0	54.2	7.1	47.1	8.5	55.0	6.6	< 0.001^b^	0.001^b^	0.83	0.514	0.17	< 0.001^b^	0.99	0.004^b^	0.92	0.617	0.13	< 0.001^b^	1.11
Caregiver-Specific Anxiety^c^	49.9	8.4	56.9	6.0	48.7	11.2	58.0	8.1	< 0.001^b^	< 0.001^b^	0.90	0.594	0.14	< 0.001^b^	0.98	0.003^b^	0.98	0.567	0.15	< 0.001^b^	1.02
Caregiving Relationship Satisfaction^c^	40.9	6.4	54.6	7.7	42.6	7.0	53.8	8.7	< 0.001^b^	< 0.001^b^	2.05	0.286	0.27	< 0.001^b^	1.78	< 0.001^b^	1.62	.706	0.10	< 0.001^b^	1.36

*N* = 328 (164 SMVs, 164 IPs): Both Satisfied = 72 dyads; SMV_sat_/IP_dis_ = 25 dyads; SMV_dis_/IP_sat_ = 21 dyads; Both Dissatisfied = 46 dyads.

*IP*, intimate partner; *Social role activities*, ability to participate in social roles and activities.

a Scores were transposed so that higher scores reflected worse functioning for all measures.

b Indicates statistical significance after controlling the false discovery rate using the Benjamini and Hochberg (33) step-down procedure to adjust the significance criterion. Cohen’s *d* effect size interpretation *d*: small, 0.2; medium, 0.5; large 0.8.

c IPs identified as not caregiving = 14.

Overall, the Benjamini and Hochberg (33) step-down procedure did not alter the significance when larger effects were observed. This applied to the majority of significant findings (see **Tables 2**–**4**). Some changes in significance were observed for smaller effect sizes. However, for many comparisons that were no longer significant after correction, a medium-large effect was observed (e.g., *d* = 0.60–0.78). Nonsignificance after correction with a medium-large effect size may due to the small sample sizes (i.e., SMV_sat_/IP_dis_, *n* = 25; SMV_dis_/IP_sat_, *n* = 21), and the findings may have remained significant with a larger sample size. While caution should be applied with multiple comparisons, it should also be applied in overlooking nonsignificant findings with medium-large effect sizes after correction is applied.

## Discussion

Approximately 40% of SMVs (40.9%) and IPs (43.3%) were dissatisfied with their relationship. Close to 30% of both members of the dyad were dissatisfied. Relationship dissatisfaction was generally associated with worse health and family outcomes. Within dyads, SMVs tended to report worse outcomes compared to their IPs, except when the SMV was satisfied and the IP was dissatisfied. Differences between couples were strongest when the IP was satisfied and the SMV was dissatisfied. The majority of SMVs and IPs agreed on their level of relationship satisfaction or dissatisfaction. When both members of the dyad agreed, there were no differences between couples on Perceived Rejection, Emotional Support, or Family Functioning measures. However, SMVs reported worse scores on the Family Experiences measure. The mixed satisfaction group, with SMV satisfied and IP dissatisfied (SMV_sat_/IP_dis_), was the only group where IPs had worse scores than their SMV. This difference was observed for the General Life Satisfaction, Perceived Rejection, Emotional Support, and Family Functioning measures, but not Family Experiences, which was the only measure where SMVsat/IPdis couples did not differ. The mixed group with the IP satisfied and SMV dissatisfied (SMV_dis_/IP_sat_) was the only group where the SMV reported worse scores on Family Functioning compared to their IP.

Across the relationship satisfaction dyad groups, dissatisfied SMVs reported worse individual and family outcomes compared to satisfied SMVs. Similarly, dissatisfied IPs reported worse outcomes compared to satisfied IPs. The effects across groups were strongest between SMVs in the Both Dissatisfied group and those in the Both Satisfied group, as well as between IPs in the Both Dissatisfied group and those in the Both Satisfied group. The effect across groups for the mixed satisfaction groups was observed only for the member of the dyad who endorsed relationship dissatisfaction. In summary, many SMVs with TBI and their IPs reported dissatisfaction in their relationship. Relationship dissatisfaction was linked to worse health and family outcomes, even if the other member of the dyad reported satisfaction in the relationship. For most comparisons, the effects were large enough to remain significant after applying Benjamini and Hochberg’s (33) step-down procedure to reduce the risk of false-positive findings from multiple comparisons.

The findings for the two family measures within dyads reveal a potentially interesting family dynamic in military couples following SMV TBI. Items on the Family Experiences measure reflect the responder’s individual relationship with their family (e.g., *My opinions are valued by other family members, I feel like I fit in with my family, I play an important role in my family*). Whereas items on the Family Functioning measure reflect how the responder feels about their family dynamic as a whole (e.g., *We are able to make decisions about how to solve problems, We confide in each other, In times of crisis we can turn to each other for support*). For SMVs, their perceptions of their individual experiences with their family tended to be worse than their perceptions of their family’s overall functioning. It is possible that the SMV’s neurobehavioral and comorbid symptoms were both influencing and being influenced by the quality of their interpersonal relationships and emotional bonds with their family. These experiences may have been more impactful than their perceptions of the dynamics and cohesiveness of their family as a whole. Neurobehavioral symptoms are less observable and, therefore, more ambiguous in their origin. Without obvious physical impairment, it can be challenging for family members to understand why the SMV is struggling to resume family roles and reconnect emotionally following a TBI, particularly when the injury is sustained during deployment and compounded by co-occurring psychological conditions (e.g., PTSD). Military families face unique stressors associated with frequent deployments, which involve lengthy periods of separation followed by reintegration. During reintegration, the family undergoes a period of re-establishing roles, responsibilities, and emotional bonds, which can be a significant source of stress for military families. Neurobehavioral changes in the service member can make the reintegration process more challenging (34–36).

For both SMVs and IPs, dissatisfaction in their relationship tended to be most strongly related to measures of relationships and social support (Perceived Rejection, Emotional Support), family (Family Functioning, Family Experiences), and general life satisfaction measures. For SMVs, PTSD cluster D also emerged as an outcome strongly related to relationship dissatisfaction. For IPs, the caregiving measures (Caregiver Strain, Caregiver-specific Anxiety, Caregiving Relationship Satisfaction) were strongly related to relationship dissatisfaction. Cluster D represents negative cognition and mood symptoms, and, of the four PTSD clusters, it has demonstrated the strongest association with relationship distress in military couples (15). SMVs experiencing negative cognition and mood symptoms can be emotionally distant and less likely to engage in intimacy and emotional communication. Emotional numbing and withdrawal symptoms tend to be nonspecific, which can lead to ambiguity in their etiology. When internalized by IPs, these symptoms may be attributed to a lack of love or the demise of the relationship, leading to elevated relationship and psychological distress. SMVs who were dissatisfied in their relationship also reported worse scores on PTSD clusters B, C, and E compared to SMVs who were satisfied. These clusters reflect agitation and irritability, distressing dreams and flashbacks, and situational avoidance. IPs often adjust their emotions and behaviors in an attempt to manage or reduce PTSD-related symptoms. These accommodative behavioral and emotional actions can lead to elevated psychological, social, caregiving, relationship, and family distress in IPs. Additionally, such accommodations can reinforce PTSD symptoms and undermine treatment and return-to-duty outcomes (15, 16).

These bidirectional and reciprocal family dynamics may have important implications for military TBI treatment programs. If a service member is discharged from treatment to a home environment with high levels of distress and dysfunction, improvement in symptoms and return-to-duty outcomes may diminish over time. Conflict, disorganization, and poor affective and behavioral regulation, along with accommodations within their family, may undermine treatment outcomes. Recent research by our team has demonstrated the strong association between family distress and chronic neurobehavioral symptoms in SMVs with TBI. Our team has shown a very strong negative association between an unhealthy family environment and SMV brain health, particularly during recovery from a TBI. We found that a SMV with a mild TBI living in an unhealthy environment was 9.8 times more likely to experience poor neurobehavioral outcomes compared to noninjured healthy controls in an unhealthy family environment, and 28.1 times more likely to have poor outcomes compared to healthy controls in a healthy family environment. Similarly, a SMV with a more severe TBI living in an unhealthy environment was 5.9 times more likely to experience poor neurobehavioral outcomes compared to healthy controls in an unhealthy family environment, and 16.9 times more likely to have poor outcomes compared to healthy controls in a healthy family environment (37). Examining the influence of IP HRQOL on SMV brain health, our team found a strong influence of IP physical, psychological, social, and caregiving HRQOL as risk factors for chronic neurobehavioral symptoms in their SMVs with TBI (37–39). Our team also found that IPs of SMVs receiving treatment at the NICoE’s IOP showed a worsening longitudinal trend in scores on many of these HRQOL risk factor measures (40).

Meta-analysis has highlighted improvement in SMVs with TBI after participating in cognitive rehabilitation (41). Some researchers have begun including intimate partners in cognitive rehabilitation programs, utilizing a couple-based or conjoint intervention design to improve both individual symptoms and relationship functioning simultaneously in military couples as a dyadic approach to treatment outcomes. *Cognitive-Behavioral Conjoint Therapy* (CBCT) is a couples therapy that employs dyadic cognitive-behavioral approaches for PTSD. CBCT has been helpful in reducing psychological and relationship distress, social and communication avoidance, and the use of accommodations in military couples with SMV PTSD (42–44). An online, guided, self-help adaptation of CBCT, *Couple Helping Overcome PTSD and Enhance Relationships* (HOPES), has also proven effective in reducing PTSD symptoms, relationship distress, and the use of accommodations (45). Dyadic cognitive-behavioral approaches may be beneficial for improving health, return-to-duty, and readiness outcomes in military couples with service member TBI.

Success has also been demonstrated in reducing individual, couple, and family-level distress in military families who participated in the *Families OverComing Under Stress* (FOCUS) family-based resilience and prevention program (46, 47). FOCUS is a training program that teaches practical skills to help military families respond to and cope with stress and changes related to military life and trauma. FOCUS is established at US military installations and also offers an interactive online platform. FOCUS may be beneficial for military families navigating neurobehavioral symptoms and treatment goals in warfighters with TBI.

In response to the growing body of research demonstrating the bidirectional associations between SMV neurobehavioral symptoms and individual, couple, and family distress in military families, the CGFM Study team recently established a Family Wellness Program (FWP) for IP beneficiaries of service members receiving treatment at the NICoE (48). IPs complete a set of measures to screen for elevated physical, psychological, social, and caregiving distress, followed by a consultation with a clinician. IPs receive a brief clinical report that includes a clinical interpretation of their symptom severity (normal, mild, moderate, severe) along with clinical recommendations such as potential treatment options, referral needs and pathways, and tailored session attendance during the family week (fourth week) of the IOP. Intimate partners are also provided with educational materials and resource guides containing information about national and local resources and programs for military families. The FWP is expanding operations across the Defense Intrepid Network (DIN) and Intrepid Spirit Centers (ISC). Inclusion of an online, self-paced, dual-goal, dyadic intervention for IPs and service members, such as the HOPES, may help maintain service member improvement in symptoms and return-to-duty outcomes, as well as IP HRQOL postdischarge from the NICoE and ISC treatment programs.

In the current study, 85% of the sample of SMVs had sustained an uncomplicated MTBI. However, the sample size of those SMVs with a more severe TBI was insufficient to compare outcomes across TBI severity groups. It is important to interpret these findings within the context of recovery from TBI and comorbidities. On average, SMVs in this sample were 12.4 years postinjury, with most having sustained an uncomplicated MTBI. Recovery from an uncomplicated MTBI is typically expected within a few weeks. In the military, comorbid conditions can confound self-reported chronic neurobehavioral symptoms, regardless of TBI severity (18). Neurobehavioral symptoms overlap with those associated with many non-TBI clinical conditions. PTSD, depression, substance use, headaches, chronic pain, and chronic headaches were prevalent comorbid conditions in the SMVs’ medical records. These comorbid conditions were likely contributing to the health and family outcomes in the current dyad sample. Previous research has shown that TBI severity had little association with individual, couple, and family-level distress in adult and child members of military families (3, 4, 49). When an effect was found, worse outcomes were reported in family members of SMVs with a MTBI compared to those with more severe TBI (3). In contrast, the SMV’s neurobehavioral symptoms have consistently been associated with individual, couple, and family-level distress in military families (4, 8, 9, 14, 50). This effect was primarily attributed to neurobehavioral symptoms related to adjustment (e.g., anxiety, depression, aggression, pain, headaches, fatigue, social, and relationships), but to a lesser extent by neurobehavioral symptoms related to ability (e.g., mobility, vision, speech, memory, attention, and concentration) (14, 50, 51). As mentioned earlier, neurobehavioral symptoms are less observable and, therefore, more ambiguous in their origin (52). A MTBI is generally associated with less observable physical and neurological impairment, thus increasing the potential for ambiguity. This ambiguity may contribute to greater disruption in family relationships. The role of TBI severity in health and family outcomes among military couples warrants further exploration.

Several potential limitations are worth mentioning. First, the directionality of the association between IP and SMV health outcomes and relationship satisfaction was not established. The relationship is likely to be an interaction of bidirectional and reciprocal individual and couple factors, where SMV neurobehavioral symptoms, IP HRQOL, and relationship satisfaction impact and are impacted by each other. Structural equation modeling or two-stage least squares would be useful to explore these reciprocal relationships. These types of multivariate structural relationships require larger sample sizes than those used in the current study. Reciprocal models also require theoretically driven instrumental variables to function mathematically. Instrumental variables need to be defined in advance, rather than identified statistically. Future research exploring dyadic relationships in military couples will contribute to the development of a theory based on these observed patterns, which can be further tested and refined using reciprocal modeling statistical methods in the future. Second, the small sample sizes in some groups (i.e., SMV_sat_/IP_dis_, *n* = 25; SMV_dis_/IP_sat_, *n* = 21) may have undermined statistical power (i.e., nonsignificant meaningful effect sizes), potentially causing true differences to be missed. The small sample sizes may also have influenced nonsignificant findings with medium-large effect sizes after controlling the false discovery rate using the Benjamini and Hochberg (33) step-down procedure to adjust the significance criterion. Third, pre-existing individual and relationship distress prior to the SMV’s TBI were not accounted for, which limits our understanding of any causal relationships. Methodologically, accruing a longitudinal sample of dyads prior to a TBI would be challenging and costly, and would heavily rely on the chance of the SMV incurring a TBI after enrolling in the study. A noninjured control cohort may be an acceptable alternative. Fourth, the majority of the sample identified as a white, non-Hispanic, female of a male SMV with an uncomplicated MTBI and of veteran status. Race and ethnicity data for the SMVs were not collected. The current study also used convenience sampling, an approach that may lack clear generalizability to the larger population due to potential bias and underrepresentation of subgroups. The findings may not be generalizable to underrepresented populations, including families still in the DoD. Finally, because military families face unique stressors (e.g., frequent relocation, deployment, reintegration), it is uncertain how generalizable the findings would be to civilian couples with TBI. Additionally, within military couples, it is not certain whether families of warfighters with combat deployments and experiences differ from military families without combat deployments and experiences. Further research is needed to determine if the findings extend to civilian and noncombat military families.

A high prevalence of SMVs with TBI and their IPs were dissatisfied with their relationship. Relationship dissatisfaction was associated with worse health and family outcomes, even if the other member in the dyad was satisfied in their relationship. Healthy relationship functioning can promote mental health treatment utilization, particularly among SMVs with greater symptom severity and average to high relationship satisfaction. Many SMVs with TBI, PTSD, and other polytrauma have expressed a preference for couples and family therapy over individual treatment (53). Couples therapy using a cognitive-behavioral conjoint approach, with the dual goals of treating individual distress and enhancing relationship functioning, has proven effective in improving the SMV’s symptoms, the IP’s symptoms, and family relationships, as well as reducing the use of negative emotional and behavioral accommodations (43, 45). A dual-goal, dyadic, and family approach to TBI treatment that emphasizes the interaction between individual, couple, and family factors will likely maximize service member recovery, return-to-duty outcomes, and outcomes for military families. The establishment of the FWP at the NICoE and expansion across the DIN will pave the way for family wellness to become a long-term component of DoD TBI treatment programs, promoting a holistic, family-centered interdisciplinary model of care that supports service member brain health, return to duty following a TBI, and the development of healthy, resilient, and mission-ready military families.

## Data Availability

The datasets presented in this article are not readily available because participants did not consent to data sharing publicly. Requests to access the datasets should be directed to Tracey Brickell, tbrickell@gdit.com.

## Author disclaimer

The views expressed in this manuscript are those of the authors and do not necessarily represent the official policy or position of the Defense Health Agency, Department of Defense, or any other US government agency. This work was prepared under Contract HT0014-22-C-0016 with the DHA Contracting Office (CO-NCR) HT0014 and is therefore defined as US Government work under Title 17 U.S.C.§101. Per Title 17 U.S.C.§105, copyright protection is not available for any work of the US Government. For more information, please contact dha.TBICOEinfo@health.mil.
